# Implementation of an Electronic Clinical Decision Support System for the Early Recognition and Management of Dysglycemia in an Inpatient Mental Health Setting Using CogStack: Protocol for a Pilot Hybrid Type 3 Effectiveness-Implementation Randomized Controlled Cluster Trial

**DOI:** 10.2196/49548

**Published:** 2024-04-05

**Authors:** Dipen Patel, Yamiko Joseph Msosa, Tao Wang, Julie Williams, Omar G Mustafa, Siobhan Gee, Barbara Arroyo, Damian Larkin, Trevor Tiedt, Angus Roberts, Richard J B Dobson, Fiona Gaughran

**Affiliations:** 1 Department of Psychosis Studies Institute of Psychiatry, Psychology and Neuroscience King's College London London United Kingdom; 2 National Institute for Health Research, Maudsley Biomedical Research Centre South London and Maudsley National Health Service Foundation Trust London United Kingdom; 3 South London and Maudsley National Health Service Foundation Trust London United Kingdom; 4 Department of Biostatistics and Health Informatics Institute of Psychiatry, Psychology and Neuroscience King’s College London London United Kingdom; 5 Centre for Implementation Science Health Service and Population Research Department King's College London London United Kingdom; 6 Department of Diabetes King's College Hospital National Health Service Foundation Trust London United Kingdom; 7 Centre for Education Faculty of Life Sciences and Medicine King’s College London London United Kingdom; 8 Institute for Health Informatics University College London London United Kingdom; 9 Health Data Research UK University College London London United Kingdom

**Keywords:** blood sugar, CDSS, clinical decision support system, decision support, diabetes, diabetic, dysglycemia, electronic clinical decision support, hyperglycemia, hypoglycemia, implementation, medical informatics, mental health, mental healthcare, mental illness, metabolic health, randomized controlled trial, RCT

## Abstract

**Background:**

Severe mental illnesses (SMIs), including schizophrenia, bipolar affective disorder, and major depressive disorder, are associated with an increased risk of physical health comorbidities and premature mortality from conditions including cardiovascular disease and diabetes. Digital technologies such as electronic clinical decision support systems (eCDSSs) could play a crucial role in improving the clinician-led management of conditions such as dysglycemia (deranged blood sugar levels) and associated conditions such as diabetes in people with a diagnosis of SMI in mental health settings.

**Objective:**

We have developed a real-time eCDSS using CogStack, an information retrieval and extraction platform, to automatically alert clinicians with National Health Service Trust–approved, guideline-based recommendations for dysglycemia monitoring and management in secondary mental health care. This novel system aims to improve the management of dysglycemia and associated conditions, such as diabetes, in SMI. This protocol describes a pilot study to explore the acceptability, feasibility, and evaluation of its implementation in a mental health inpatient setting.

**Methods:**

This will be a pilot hybrid type 3 effectiveness-implementation randomized controlled cluster trial in inpatient mental health wards. A ward will be the unit of recruitment, where it will be randomly allocated to receive either access to the eCDSS plus usual care or usual care alone over a 4-month period. We will measure implementation outcomes, including the feasibility and acceptability of the eCDSS to clinicians, as primary outcomes, alongside secondary outcomes relating to the process of care measures such as dysglycemia screening rates. An evaluation of other implementation outcomes relating to the eCDSS will be conducted, identifying facilitators and barriers based on established implementation science frameworks.

**Results:**

Enrollment of wards began in April 2022, after which clinical staff were recruited to take part in surveys and interviews. The intervention period of the trial began in February 2023, and subsequent data collection was completed in August 2023. Data are currently being analyzed, and results are expected to be available in June 2024.

**Conclusions:**

An eCDSS can have the potential to improve clinician-led management of dysglycemia in inpatient mental health settings. If found to be feasible and acceptable, then, in combination with the results of the implementation evaluation, the system can be refined and improved to support future successful implementation. A larger and more definitive effectiveness trial should then be conducted to assess its impact on clinical outcomes and to inform scalability and application to other conditions in wider mental health care settings.

**Trial Registration:**

ClinicalTrials.gov NCT04792268; https://clinicaltrials.gov/study/NCT04792268

**International Registered Report Identifier (IRRID):**

DERR1-10.2196/49548

## Introduction

### Overview

People with severe mental illnesses (SMIs) such as schizophrenia, bipolar affective disorder, and schizoaffective disorders have a significantly reduced life expectancy in comparison with the general population. These groups have higher rates of cardiovascular disease (CVD) risk factors such as central obesity, high blood pressure, raised cholesterol levels, and raised blood sugar levels compared with the general population [1,2].

Improvements to the primary prevention of diabetes in the general population have not been replicated to the same degree in people with SMI [3]. Diabetes refers to a group of metabolic disorders characterized by a high blood sugar level (dysglycemia) over an extended period of time [4]. If unrecognized, untreated, or poorly managed, diabetes can lead to long-term health complications, including CVD, stroke, chronic kidney disease, foot ulcers, retinopathy, and peripheral neuropathy [4,5].

Diabetes accounts for approximately 10% of health care resources in the United Kingdom, and this is set to rise to 17%, with total costs of GBP 39.8 (US $50.7) billion estimated by 2035, when direct health care costs and indirect costs on productivity are taken into account [6]. Recorded rates of diabetes among ethnically diverse middle-aged people with a diagnosis of established psychosis in South London reach 20%, with a further 30% evidencing raised blood sugar levels (dysglycemia) [7]. Likewise, the prevalence of both diabetes and dysglycemia is higher in inpatient psychiatric settings than in the general community [8]. Furthermore, rates of dysglycemia double in the first year after a first psychotic episode, creating a unique window for prevention strategies to address these risks as early as possible [9].

Diabetes outcomes are poor in SMI groups, with people with schizophrenia and cooccurring diabetes having an increased risk of excess mortality, including postcomplication mortality [10]. A key inequality affecting people with SMI is the less than–adequate assessment and management of physical health conditions such as diabetes. In order to target the physical health care of people with SMI and close the life expectancy gap, a number of evidence-based clinical guidelines and policies have been published over the past decade [11-13].

Unfortunately, there remains significant variation in the implementation of these guidelines and recommendations in mental health care services, as outlined by the National Audit of Schizophrenia [14]. A retrospective audit of people diagnosed with schizophrenia or schizoaffective disorder revealed that among those with dysglycemia, only 53.5% were recorded as receiving an appropriate intervention, and among those with dyslipidemia, this was only 19.9% [15]. Another study found that people with SMI and diabetes were not receiving the expected standards of care in glucose monitoring or access to specialist diabetes services when admitted to a psychiatric unit [16].

Globally, studies evaluating the provision of care by clinicians reveal a suboptimal uptake of clinical guidelines into practice. The underlying reasons for this are complex and noted to occur at a combination of patient, clinician, and wider systemic levels [17]. There is therefore a need for more targeted and clinically informed interventions that improve the standard of physical health care screening and interventions offered to people with SMI across both primary and secondary care settings.

Digital health solutions have the potential to improve the delivery of care through tools such as clinical decision support systems [18]. An electronic clinical decision support system (eCDSS) consists of digital-enabled tools and interventions [19,20] that can be “based on a software algorithm designed to aid directly in clinical decision-making, in which characteristics of individual patients are used to generate patient-specific assessments or recommendations that are then presented to clinicians for consideration” [19]. An eCDSS has the potential to overcome problems associated with the use and implementation of traditional paper-based guidelines. Interventions involving clinical decision support systems appear to achieve small to moderate improvements in targeted processes of care in acute physical health care settings [21]. However, the evidence base for eCDSSs in mental health care settings remains sparse.

Given the high disease burden of diabetes in SMI and the deficits in providing evidence-based care for diabetes prevention and treatment, there is a pressing need to identify novel systems-focused solutions. The adoption of digital technology to improve physical health in people with a diagnosis of SMI presents a unique opportunity but requires evidence of acceptability, feasibility, and effectiveness.

Digital systems that are not accepted by their users cannot be expected to contribute to improving the quality of care; hence, facilitators, barriers, and other unintended consequences need to be understood for the successful implementation of novel digital tools and could also serve as a basis for future system reengineering. Hence, there is a call for research to include evaluating its implementation to improve the chances of successful future scalability [22].

Given that patients with SMI have a high risk of CVD factors, including dysglycemia and diabetes, and that there is typically a suboptimal uptake of clinical guidelines, there is a need for more targeted and clinically informed interventions that improve the standard of physical health care screening and interventions offered to people with SMI. Assessing the physical health of patients with SMI when they are admitted under mental health services offers an opportunity to identify risk factors for developing conditions such as CVD or diabetes and provide advice and support to their care teams on services that can be accessed in hospitals and in the community.

We have previously reported on the design and development of a novel eCDSS built using the information retrieval and extraction platform CogStack, deployed at the South London and Maudsley National Health Service (NHS) Foundation Trust, UK (CogStack@Maudsley), comprising a real-time computerized alerting and clinical decision support system for dysglycemia management that has been previously validated for use in secondary mental health care [23].

### Objectives

The primary objective of this study is to establish the acceptability, feasibility, and other implementation challenges of the eCDSS (CogStack@Maudsley), comprising a real-time computerized alerting and clinical decision support system, in supporting dysglycemia management in an inpatient secondary mental health care setting.

Our secondary objective is to assess the change in rates of guideline-indicated tests or interventions for dysglycemia on the test wards before and after the introduction of the eCDSS, compared to comparator wards without access to the eCDSS.

This will be measured using pseudonymized group observational data gathered from the South London and Maudsley NHS Foundation Trust Biomedical Research Centre’s Clinical Records Interactive Search (CRIS) system. Since 2006, the South London and Maudsley NHS Trust has operated fully electronic health records. The CRIS system, established in 2008, is an ethically approved electronic health records interface system that allows researchers to access deidentified electronic health records from this trust for research purposes [24-26].

Data gathered from this study will allow us to refine the system, address potential challenges with future successful implementation, and inform a larger and more definitive effectiveness trial that will examine for hypothesized improvements in (1) rates of clinician-delivered, evidence-based interventions for patients with dysglycemia and (2) clinical outcomes relating to diabetes care.

## Methods

### Study Design

We will conduct a pilot hybrid type 3 effectiveness-implementation randomized controlled cluster trial in inpatient mental health ward settings [27]. This specific study design was chosen as the primary aim is focused on implementation outcomes, while the secondary aims relate to effectiveness outcomes. Wards will be the unit of recruitment and will be assigned to deliver care with either the eCDSS platform alongside usual care processes (treatment as usual [TAU]) or to follow TAU alone. The trial will last for a period of 4 months. Qualitative implementation outcome data will be obtained from participating clinicians on all recruited wards using implementation pre- and poststudy surveys and individual semistructured interviews. Quantitative indicative effectiveness data to inform future work will be gathered using pseudonymized group data, including rates of adherence to dysglycemia guidelines and process of care measures.

### Setting

The study will be conducted with clinicians in 5 general adult psychiatry inpatient wards at South London and Maudsley NHS Foundation Trust in the United Kingdom.

### Recruitment and Eligibility

Screening for dysglycemia is indicated in all people admitted to general adult inpatient wards at the host NHS Trust hospital. All general adult inpatient wards in the hospital will be eligible for inclusion in the study, with the study publicized at consultant continuing professional development meetings and to senior management of all wards at the trust. Managers of wards who show an initial interest will be approached and their wards invited to participate until a total of 5 participating wards is reached. All clinicians on participating wards who are routinely involved in dysglycemia management, including consultant psychiatrists, medical doctors, ward pharmacists, and the ward nursing team, will be eligible and invited to consent to participate in the preintervention surveys and interviews and postintervention surveys and interviews if they were on an intervention ward at the end of the study. 

Processes of care in each participating ward will be obtained using the available pseudonymized observational data of all people receiving inpatient care in participating wards during the trial period. Individual patient outcomes will not be recorded.

### Randomization

Wards will be the unit of recruitment and will be assigned randomly to either the intervention or TAU group to receive either the eCDSS platform alongside usual care or to follow usual care processes only for a period of 4 months. We will recruit and randomly allocate 2 wards to the intervention arm and 3 wards to the comparator arm using fixed block randomization. Random allocation of the wards will be done by an independent trial manager who is outside of the research team to ensure there is no bias in allocations.

### Sample Size

The primary end points of this study are implementation outcomes rather than direct measures of intervention effects on clinical outcomes. Hence, power analyses for intervention outcomes have not been undertaken in advance. A total of 5 wards will be recruited in total, which will be a large enough sample of wards to recruit enough staff to take part in preintervention qualitative work, and to inform the practicalities of implementing the system and collecting sufficient outcome data. 

### Intervention

We previously developed an eCDSS for dysglycemia hosted within CogStack@Maudsley [23]. The eCDSS is designed to alert clinicians automatically when patients are admitted under their care regarding the need for screening for, or management of, dysglycemia. Prompts are triggered by the presence of new, old, or absent HbA_1c_ pathology reports in the electronic health record.

Digital-enabled clinical decision support is provided as a combination of visual prompts on a dashboard embedded within an existing electronic platform used at the NHS Trust and email supplements sent to NHS Trust email accounts of participating wards. The eCDSS was designed to integrate as easily as possible within existing workflows by building it into the existing NHS Trust electronic systems that clinicians use in inpatient wards.

The alerts in this study will include NHS Trust “Quality Centre”–approved, guideline-based recommendations for clinician-led monitoring and management of dysglycemia and known diabetes, tailored to the individual patient’s reported HbA_1c_ values. The eCDSS algorithm is based on gold-standard clinical management guidelines, which were developed and agreed upon by the NHS Trust [23].

Clinicians on intervention wards will be able to view priority-tagged prompts in the EHR system, corresponding to guideline-based recommended actions they can access for further patient-specific guidance with regard to their care. Clinicians are already familiar with the platform for other uses, but a demonstration of the system will be given to users before the intervention period, so they are aware of how the system is accessed and used. Access to the system will be restricted to the intervention wards only, but access to it can be expanded in the future to more wards and teams.

The research team will be available to provide support to clinicians participating in the study to ensure that any technical queries relating to the eCDSS are dealt with appropriately. The research team will also work closely with the host trust IT department so that any potential technical issues are responded to quickly.

### Study Procedure

Ward managers of the selected wards will notify ward staff of the study and the opportunity to participate in interviews and surveys as part of this study. All clinical staff working on recruited wards who express an initial interest to their ward manager will be approached by members of the research team and given a participant information leaflet and an opportunity to ask and discuss any further questions regarding the study. They will be invited to take part in an initial survey and an individual interview with a member of the research team at the start of the study, and then again at the end of the trial, should their ward be randomized to the intervention arm.

Consented participants will be invited to complete an initial survey and to take part in an individual interview lasting approximately 30 minutes. The interview will follow a semistructured interview topic guide with key prompts to direct the discussion, but researchers will be able to direct further questions if additional themes or content arise during the course of the interview. The survey and interview aim to scope the experiences of clinicians in managing diabetes and related physical health conditions in secondary mental health care settings and to explore their attitudes toward the use of novel digital technology as a means of improving physical health care provision in secondary mental health care settings.

Participants in the intervention arm of the study will be invited, a minimum of 4 months from the start of the study, to complete a follow-up survey and individual interview lasting approximately 30 minutes, which will aim to scope their experiences and attitudes toward using the eCDSS and their perceptions of its impact upon diabetes and dysglycemia care. The interview will again follow a semistructured interview topic guide with key prompts to direct the discussion, but researchers will be able to direct further questions if additional themes or content arise during the course of the interview.

Service-level process of care outcome data will be collected at the level of participating wards using pseudonymized observational group-level data from the CRIS system [25]. Approvals for the acquisition of relevant deidentified data through the CRIS system will be applied for through the CRIS oversight committee.

### Ethical Considerations

This study was reviewed and approved by the NHS Health Research Authority and Health and Care Research Wales (reference 285509). The eCDSS has received NHS digital clinical safety sign-off by the host trust, and any immediate patient safety issues will be reported in line with the digital safety policy at the host NHS Trust. The eCDSS is built within existing IT systems at the trust; therefore, data do not leave the firewall of the existing systems.

Each participant will be given an identification number. All information collected will be kept confidential; all identifiable data will be securely stored; and forms with identifiable data, such as consent forms, will be kept separate from the outcome data in a locked cupboard. All data generated will be stored securely, such that participants can only be identified by their unique study identifier, and all stored electronic data will be password-protected.

### Outcomes and Data Collection

As this is a feasibility study, the primary outcome relates to acceptability, feasibility, and other implementation outcomes relating to the intervention (Table 1). Secondary outcomes relate to the process of care measures (ie, screening rates for dysglycemia, documentation of interventions relating to dysglycemia and diabetes care, and documentation of communication to other relevant care professionals regarding follow-up). The study is not powered to detect impacts on clinical outcomes. As this is a pilot study, the primary outcomes will be used to inform further work to refine the system before progressing to a full trial.

**Table 1:** Outcome measures.

Outcomes			Definition		Data collection
**Primary outcomes (implementation outcomes)**					
	Acceptability	Extent to which eCDSS^a^ is perceived by clinical users to be acceptable in prompting evidence-based dysglycemia management, and an effective system for improving dysglycemia and, where applicable, diabetes care. Experience of the system Effect on workload Barriers and facilitators to its use Impact on clinicians’ time compared to usual care Perceived effectiveness of the system Intention to continue to use the system		Qualitative: pre- and postsurvey and semistructured individual interviews	
	Feasibility	Ability to recruit wards and clinicians to the study. Retention and participation of clinicians on recruited wards through to the end of the study. Usefulness and limitations of CRIS to collect process of care outcome measures		Quantitative and qualitative: survey and semistructured individual interviews	
	Adoption	Individual clinicians and the wider system’s intention to adopt and use the system for dysglycemia management.		Qualitative: survey and semistructured individual interview	
	Reach	Number of clinicians who make use of the system as a proportion of the total number of clinicians expected to use the system		Qualitative: survey and semistructured individual interview Quantitative: audit trail on eCDSS platform	
	Appropriateness	Extent to which the novel system is perceived to be fit and relevant for dysglycemia management by users		Qualitative: survey and semistructured individual interview	
	Fidelity of Delivery	Extent to which the eCDSS system alerts are delivered as intended.		Qualitative: survey and semistructured individual interview Quantitative: eCDSS platform	
	Sustainability	Facilitators and barriers to sustained use of the system. Clinician attitudes toward the system.		Qualitative: survey and semistructured individual interview	
	Contextual Factors	Facilitators and barriers to the implementation of the system		Qualitative: survey and semistructured individual interview	
	Maintenance	Satisfaction of, and intention to use eCDSS, by clinicians		Qualitative: survey and semistructured individual interview	
**Secondary outcomes (processes of care)**					
	HbA_1c_^b^ testing	Rates of HbA_1c_ testing on inpatients		Quantitative	
	Documentation of dysglycemia or diabetes in clinical notes	Documentation of diabetes or prediabetes diagnosis in case notes during inpatient stay (where indicated)		Quantitative	
	Documentation of discussion with patient regarding exercise, diet, and smoking cessation	Documentation of advice by clinician given to the patient regarding lifestyle changes—exercise, diet, and smoking cessation in patients with dysglycemia		Quantitative	
	Documentation of diabetes-related screening interventions	Documentation of completed foot check for patients with diabetes		Quantitative	
	Delivery of evidence-based pharmacological interventions for diabetes or prediabetes where clinically indicated	Documentation of discussion regarding diabetes-related medication changes post alerting where clinically indicated: Initiation of diabetes-related medication Intensification of medication (dose change or introduction of new agent in accordance with guidance)		Quantitative	
	Communication with general practitioner or community mental health team regarding diabetes or dysglycemia follow-up	Documentation to relevant community teams and GP^c^ regarding follow-up plans for dysglycemia management post discharge where indicated		Quantitative	

^a^eCDSS: electronic clinical decision support system.

^b^HbA_1c_: glycated hemoglobin.

^c^GP: general practitioner.

A process evaluation of the implementation of the intervention will be conducted to identify factors that inhibit or facilitate implementation. A mixed methods approach will be adopted to establish feasibility, acceptability, and other implementation outcomes.

Details of the study were first shared among ward managers and consultants in January 2022, and formal enrollment of eligible wards began in April 2022. Clinical staff on recruited wards were then individually recruited to take part in surveys and interviews starting in June 2022. The intervention period of the trial began in February 2023, and qualitative data collection was completed in August 2023. All interviews were recorded and subsequently transcribed verbatim.

### Baseline Measurements

We will collect the following sociodemographic data on participating clinicians from recruited wards: age, gender, years of clinical experience, specialty or profession, and grade.

No identifiable data will be collected on patients on the recruited wards. However, a separate application will be made to the CRIS oversight committee to gather the following pseudonymized information at a ward or group level: age, gender, ethnicity, HbA_1c_ levels, recorded history of diabetes, International Classification of Diseases-10th revision (ICD-10) mental disorder diagnosis, and history of referral to diabetes specialist teams.

### Analysis

Mixed methods will be used to analyze the outcome data generated.

Qualitative analysis of data generated through semistructured interviews and surveys of clinician users will be used to evaluate the primary outcome measures relating to implementation. A thematic analysis of interview transcripts will be conducted. We will then use a qualitative process evaluation, grounded within established implementation science frameworks, to analyze and report on the implementation outcomes generated [28-30].

We will compare changes in the process of care measures between intervention and control groups to evaluate the eCDSS impact on the process of care measures by analyzing group-level pseudonymized observational data through the CRIS platform. Descriptive statistics will be used to report on these results.

Key stakeholders will be informed of the outcomes of the study through internal report and the presentation of the study results at local meetings. It is planned that the methods and findings of the study will be incorporated into an original research paper that can be widely shared, following peer review in a journal.

## Results

As this paper is a study protocol, no results are currently available. Enrollment of wards began in April 2022, after which clinical staff were recruited to take part in preintervention surveys and interviews. The intervention period of the trial began in February 2023, and subsequent data collection for postintervention interviews and surveys was completed in August 2023. Data are currently being analyzed, and results are expected to be available in June 2024.

## Discussion

### Overview

To our knowledge, this will be the first-ever trial of an eCDSS comprising an automated electronic physical health monitoring and alerting platform developed for use in a secondary care mental health setting, which aims to improve the clinician-led management of dysglycemia and diabetes care to patients with SMI. eCDSS has the potential to radically improve the standards of physical health care offered to patients with SMI and may be a crucial step in reducing the morbidity and mortality associated with chronic physical health conditions in SMI.

Implementation of eCDSS for use in clinical settings has previously been shown to be time-consuming, and its complexity can result in slow adoption into practice [20]. To bridge this gap, we have adopted a stepwise approach, starting with technical development and in silico evaluation [23], and progressing to this pilot, with the aim of informing further refinement, development, and engineering of the eCDSS, to enable its longer-term successful adoption in clinical practice.

Integration of eCDSS into existing clinical workflows is not simple, and digital health systems that are not wholly accepted by their users cannot be expected to contribute to improving care, hence the need for research into facilitators and barriers to adoption. These need to be well understood to support the successful implementation of novel digital tools, with calls for research to include evaluating eCDSS implementation for successful future scalability [22].

Gaining a good understanding of the factors that affect the adoption and integration of digital health tools into routine practice could also serve as a basis for creating frameworks for delivering future impactful digital tools. For this reason, we have included implementation outcomes alongside other measures of effectiveness, to inform further work that aims to facilitate smoother implementation of digital technologies into clinical settings. This knowledge can then be used to apply and scale the eCDSS for other physical health conditions in SMI, such as atrial fibrillation, hypertension, and hypercholesterolemia.

This study does have some limitations. Recruitment is limited to 1 NHS Trust, with 2 wards allocated to the intervention arm from 1 site, thus limiting the extent to which broader conclusions can be made as the findings are not generalizable. There could be a self-selection bias in regard to staff who agree to participate in interviews and surveys, as it is possible that they are more likely to be interested in or accustomed to novel digital technologies. All staff will be encouraged to ask the research team about any concerns they may have so that they can be addressed with a view to reducing the risk of this. It is possible that different data might be generated if it were conducted in settings across multiple sites. Given the study design, there is also the possibility of the Hawthorne effect, which is well documented in research, when participants may knowingly or unknowingly alter their behaviors as a result of being observed as part of the study [28]. Nonetheless, this pilot study still serves as a useful basis for preliminary research in a setting that has not been explored in depth previously.

We have previously demonstrated that it is technically feasible to design and deploy a functional monitoring and alerting eCDSS for dysglycemia in a secondary care setting for mental health [23]. This pilot feasibility trial seeks to further improve our understanding of the implementation challenges of implementing a new digital system that aims to improve physical health care provision to patients in mental health settings and inform a further, more comprehensive evaluation of the eCDSS. Key implementation outcomes, including acceptability and feasibility, will be evaluated alongside outcomes relating to impacts on care processes and based on established implementation science methods [29-31].

### Conclusion

An eCDSS can have the potential to improve clinician-led management of dysglycemia in inpatient mental health settings. If found to be feasible and acceptable in this study, then, in combination with the results of the implementation evaluation, the system can be refined and improved to support future successful implementation. A larger and more definitive effectiveness trial should then be conducted to assess its impact on clinical outcomes over a longer period of time and to guide its scalability and application to other conditions in wider mental health care settings.

## Abbreviations

CRIS: Clinical Records Interactive Search
CVD: cardiovascular disease
eCDSS: electronic clinical decision support system
ICD-10: International Classification of Disease-10th revision
NHS: National Health Service
SMI: severe mental illness
TAU: treatment as usual

## Data Availability

Data sharing is not applicable to this article as no data sets were generated or analyzed during this study.
